# Supplementary material for: Safety assessment of obinutuzumab: Real-world adverse event analysis based on the FAERS and JADER databases from 2013 to 2025
Source: PLoS One. 2025 Oct 10;20(10):e0334317. doi: 10.1371/journal.pone.0334317 (PMC12513595; doi:10.1371/journal.pone.0334317)
Supplement: S1 Table — ROR, reporting odds ratio; PRR, proportional reporting ratio; CI, confidence interval; χ2, chi-squared; IC, information component; IC025, the lower bound of 95% CI; EBGM, empirical Bayesian geometric mean; EBGM05, the lower bound of 95% CI. (DOCX) [file pone.0334317.s001.docx]

**S1 Table. Four methods formula and threshold value**

| Merhod | Formula | Threshold |
| --- | --- | --- |
| ROR | $ROR = \frac{a/c}{b/d}$  $SE(lnROR) =\sqrt{\frac{1}{a}+\frac{1}{b}+\frac{1}{c}+\frac{1}{b}}$  ${95\%CI = e}^{ln(ROR)\pm1.96se}$ | a≥3  ROR≥3 95%CI> 1 |
| PRR | $PRR = \frac{a(a+b)}{c(c+d)}$  $SE(lnPRR)=\sqrt{\frac{1}{a}-\frac{1}{a+b}+\frac{1}{c}-\frac{1}{c+b}}$  ${95\%CI =e}^{ln(PRR)\pm1.96se}$  $x^{2}=\frac{(ad-bc)^{2}(a+b+c+d)}{(a+b)(a+c)(c+d)(b+d)}$ | a≥3  PRR≥２  $x^{2}$≥4 |
| BCPNN | $IC = \log_{2}\frac{p(x,y)}{p(x)p(y)} = \log_{2}\frac{a(a+b+c+d)}{(a+b)(a+c)}$ $E(IC) = \log_{2}\frac{(a+\gamma11)(a+b+c+d+\alpha)(a+b+c+d+\beta)}{(a+b+c+d+\gamma)(a+b+\alpha1)(a+c+\beta1)}$ $\begin{aligned} V(IC) = \frac{1}{(ln2)^{2}} \{\left[ \frac{(a+b+c+d)-a+\gamma-\gamma11}{(a+\gamma11)(1+a+b+c+d+\gamma)} \right]+\left[ \frac{(a+b+c+d)-(a+b)+a-\alpha1}{(a+b+\alpha1)(1+a+b+c+d+\alpha)} \right]+ \end{aligned}$ $\left[ \frac{(a+b+c+d)-(a+c)+\beta-\beta1}{(a+c+\beta1)(1+a+b+c+d+\beta)} \right]$}  $\gamma= \gamma11\frac{(a+b+c+d+\alpha)+(a+b+c+d+\beta)}{(a+b+\alpha1)(a+c+\beta1)}$  $IC-2SD = E(IC)-2\sqrt{V(IC)}$ | IC025>0 |
| EBGM | $\mathrm{EBGM}= \frac{a(a+b+c+d)}{(a+c)(a+b)}$  $SE(lnEBGM) =\sqrt{\frac{1}{a}+\frac{1}{b}+\frac{1}{c}+\frac{1}{b}}$  ${95\%CI = e}^{ln(EBGM)\pm1.96se}$ | EBGM05>2 |

ROR, reporting odds ratio; PRR, proportional reporting ratio; CI, confidence interval; χ^2^, chi-squared; IC, information component; IC025, the lower bound of 95% CI; EBGM, empirical Bayesian geometric mean; EBGM05, the lower bound of 95% CI.
