# Supplementary material for: Safety assessment of obinutuzumab: Real-world adverse event analysis based on the FAERS and JADER databases from 2013 to 2025
Source: PLoS One. 2025 Oct 10;20(10):e0334317. doi: 10.1371/journal.pone.0334317 (PMC12513595; doi:10.1371/journal.pone.0334317)
Supplement: S1 File — (DOCX) [file pone.0334317.s002.docx]

import pandas

import math

def getPRR(a, b, c, d):

if a == 0 or b == 0 or c == 0 or d == 0:

return 0

else:

return (a/float(a+b)) / (c/float(c+d))

def getROR(a, b, c, d):

if a == 0 or b == 0 or c == 0 or d == 0:

return [0, 0, 0]

else:

ROR = (a/float(c)) / (b/float(d))

try:

UpperCI = math.exp( math.log(ROR) + 1.96*math.sqrt( 1/float(a) + 1/float(b) + 1/float(c) + 1/float(d) ) )

LowerCI = math.exp( math.log(ROR) - 1.96*math.sqrt( 1/float(a) + 1/float(b) + 1/float(c) + 1/float(d) ) )

return [ROR, LowerCI, UpperCI]

except:

return [ROR, False, False]

def getχ2(a, b, c, d):

if a == 0 or b == 0 or c == 0 or d == 0:

return 0

else:

return ((a*d-b*c)**2)*(a+b+c+d) / ((a+b)*(c+d)*(b+d)*(a+c))

def getBCPNN(a, b, c, d):

if a == 0 or b == 0 or c == 0 or d == 0:

return [0, 0, 0]

else:

IC = math.log(a * (a + b + c + d) / ((a + b) * (a + c))) / math.log(2)

if IC <=0:

return [IC, False, False]

else:

variance = (1 / a + 1 / b + 1 / c + 1 / d)

if variance <= 0:

return [IC, False, False]

else:

IClower = math.exp(math.log(IC) - 1.96 * (1 / a + 1 / b + 1 / c + 1 / d)**0.5)

ICupper = math.exp(math.log(IC) + 1.96 * (1 / a + 1 / b + 1 / c + 1 / d)**0.5)

return [IC, IClower, ICupper]

def getMGPS(a, b, c, d):

if a == 0 or b == 0 or c == 0 or d == 0:

return [0,0,0]

else:

EBGM = a * (a + b + c + d) / (a + c) / (a + b)

if EBGM <=0:

return [EBGM, False, False]

else:

variance = (1 / a + 1 / b + 1 / c + 1 / d)

if variance <= 0:

return [EBGM, False, False]

else:

EBGMlower = math.exp(math.log(EBGM) - 1.96 * (1/a + 1/b + 1/c + 1/d)**0.5)

EBGMupper = math.exp(math.log(EBGM) + 1.96 * (1/a + 1/b + 1/c + 1/d)**0.5)

return [EBGM, EBGMlower, EBGMupper]
