# Supplementary material for: Safety assessment of obinutuzumab: Real-world adverse event analysis based on the FAERS and JADER databases from 2013 to 2025
Source: PLoS One. 2025 Oct 10;20(10):e0334317. doi: 10.1371/journal.pone.0334317 (PMC12513595; doi:10.1371/journal.pone.0334317)
Supplement: Attachment — Submitted filename: Response to the academic editor and reviewers.docx [file pone.0334317.s007.docx]

**Respond to** **academic editor**

1. Please ensure that your manuscript meets PLOS ONE's style requirements, including those for file naming. The PLOS ONE style templates can be found at

https://journals.plos.org/plosone/s/file?id=wjVg/PLOSOne_formatting_sample_main_body.pdf and

https://journals.plos.org/plosone/s/file?id=ba62/PLOSOne_formatting_sample_title_authors_affiliations.pdf.

Answer: Thank you very much for your valuable comments. We have revised the manuscript format as per the requirements.

2. Please note that PLOS One has specific guidelines on code sharing for submissions in which author-generated code underpins the findings in the manuscript. In these cases, we expect all author-generated code to be made available without restrictions upon publication of the work. Please review our guidelines at https://journals.plos.org/plosone/s/materials-and-software-sharing#loc-sharing-code and ensure that your code is shared in a way that follows best practice and facilitates reproducibility and reuse.

Answer: The code has been uploaded as a supplementary information file (S1 File).

3. We note that your Data Availability Statement is currently as follows: [All relevant data are within the manuscript and its Supporting Information files]

Please confirm at this time whether or not your submission contains all raw data required to replicate the results of your study. Authors must share the “minimal data set” for their submission. PLOS defines the minimal data set to consist of the data required to replicate all study findings reported in the article, as well as related metadata and methods (https://journals.plos.org/plosone/s/data-availability#loc-minimal-data-set-definition).

For example, authors should submit the following data:

- The values behind the means, standard deviations and other measures reported;

- The values used to build graphs;

- The points extracted from images for analysis.

Authors do not need to submit their entire data set if only a portion of the data was used in the reported study.

If your submission does not contain these data, please either upload them as Supporting Information files or deposit them to a stable, public repository and provide us with the relevant URLs, DOIs, or accession numbers. For a list of recommended repositories, please see https://journals.plos.org/plosone/s/recommended-repositories.

If there are ethical or legal restrictions on sharing a de-identified data set, please explain them in detail (e.g., data contain potentially sensitive information, data are owned by a third-party organization, etc.) and who has imposed them (e.g., an ethics committee). Please also provide contact information for a data access committee, ethics committee, or other institutional body to which data requests may be sent. If data are owned by a third party, please indicate how others may request data access.

Answer: The original data have been supplied as separate files within the Supporting Information files.

**Respond to Reviewer 1**

Reviewer #1: The authors have systematically and rigorously analyzed the adverse effects (AE)caused by obinutuzumab using two large (FAERS and JADER) databases.

The paper provides an interesting compilation of the drug's main AE. Considering the increasingly widespread use of this drug, it will be useful for the reader.

To enhance the interest of the paper, I suggest the following points:

1. AE may have a greater or lesser incidence and severity depending on the pathology being treated. This last aspect (indications) is poorly developed in the manuscript. They are only briefly discussed in the section about baseline characteristics, and the 5 main indications (CLL, follicular lymphoma, B-cell lymphoma, etc.) are presented in Table 3. However, these 5 top indications include “prescriptions for unknown causes” and "Other." It would be helpful to show the most common indications within the group “Other”.

Answer: Thank you very much for your valuable comments. We have raised the number of indications to 20.

2. Among the indications for obinutuzumab, its use in rheumatologic and systemic diseases will become increasingly important, given its greater efficacy than rituximab (e.g., lupus nephritis). It would be interesting to compare the frequency, characteristics, and severity of AE in hematologic malignancies (CLL, follicular or B-cell lymphomas, etc.) with non-tumoral indications.

Answer: As depicted in Figures 4 and 5, we supplemented the disparities in AEs induced by obinutuzumab in tumor and non-tumor indications.

3. In relation to the two previous points, the interest of this paper would be enhanced by analyzing AE of a recently published large trial of obinutuzumab in lupus nephritis (REGENCY; NEJM 2025; 392: 1471-83), using the same methodology applied in this paper and comparing them with the AE observed in other pathologies.

Answer: As the method employed in this article is not applicable to the large trial (REGENCY; NEJM 2025; 392: 1471-83), in Section 4.7, we have supplemented the relevant content regarding the efficacy and safety of obinutuzumab in lupus nephritis.

**Respond to Reviewer 2**

Reviewer #2: The authors conduct a large-scale, real-world pharmacovigilance analysis of adverse events (AEs) associated with obinutuzumab by analyzing the FAERS and JADER databases. They apply multiple signal detection algorithms (ROR, PRR, BCPNN, EBGM) to identify risk signals that are not currently documented in the product label. The study addresses a relevant clinical issue, post-marketing surveillance of monoclonal antibody therapies, and provides real-world evidence. However, the current manuscript lacks critical depth in interpretation, reproducibility, and clarity of English language, which limits its impact.

Main comments:

In my opinion, the study demonstrates insufficient depth in the interpretation of the data:

The authors mainly report signal values without providing a clinical context for many of the findings.

A more in-depth discussion is needed on how these adverse events may arise from a mechanistic point of view and how they compare with similar monoclonal antibodies (rituximab).

I recommend including a meta-analysis or comparative safety analysis with the existing literature to strengthen the conclusions, which appear to be only comments or opinions without scientific rigor.

Answer: Thank you very much for your valuable comments. In the discussion section, we explored the mechanisms underlying various AEs induced by obinutuzumab. Moreover, in sections 4.2 and 4.3, a meta-analysis was cited to compare the efficacy and safety differences between obinutuzumab and rituximab.

Language and grammar require revision

The manuscript contains multiple grammatical and structural errors and variations that affect readability. A thorough review by a native English speaker is recommended.

Answer: We have revised the language and grammar throughout the manuscript.

Lack of methodological transparency

The search criteria and exact filters used for FAERS and JADER are not clearly described. Without this information, the analysis is not reproducible. I recommend including a flow chart showing the number of reports included/excluded.

Answer: We have added a flow chart (Fig 1).

Does not control for confounding or bias in spontaneous reports:

The study should better acknowledge and discuss the limitations of spontaneous reports, including under-reporting, duplicate reports, and lack of denominator data. Consider incorporating a refined risk signal strategy or validating signals with external databases.

Answer: In section 4.8, we conducted a more in-depth analysis of the limitations of spontaneous reporting and data statistics.

Clarify statistical methods:

Formulas and thresholds are included, but justification for the choice of methods is lacking. Statistical corrections are also missing.

Answer: We have provided the rationale for the selection of methods in section 2.2. The reported statistics of adverse events from the FAERS and JADER databases are not appropriate for statistical corrections.

Weak conclusions and no meaningful clinical recommendations: The current conclusion merely repeats the results. Provide clinical recommendations (follow-up needs, high-risk patient profiles) and discuss implications for prescribing.

Answer: We have incorporated clinical recommendations and deliberated on the implications for prescribing within the conclusion.

Consider moving the technical descriptions of the algorithms (i.e., BCPNN formulas) to the supplementary material to improve the flow of the manuscript.

Answer: We have moved the technical descriptions of the algorithms to the supplementary material.

Tables 4 and 5 are dense and difficult to interpret. Highlight their content more clearly.

Answer: We have made revisions and improvements to Tables 4 and 5 to enhance comprehensibility.

Abbreviations (NHL, ... see text) should be defined consistently the first time they are used.

Answer: These problems have been rectified.

There are several repeated references to “not registered in the specification”; clearly define what “specification” refers to.

Answer: We have revised the “specification” to “drug instruction”.
